# AGCECDA: attention-guided heterogeneous graph collaborative embedding for circRNA–drug sensitivity association prediction

**DOI:** 10.1186/s12915-026-02656-x

**Published:** 2026-06-29

**Authors:** Chao Cao, Mengli Li, Maozu Guo, Chunyu Wang, Quan Zou, Mengting Niu

**Affiliations:** 1 Institute of Fundamental and Frontier Sciences, University of Electronic Science and Technology of China, Chengdu, Sichuan 611731 China; 2 Yangtze Delta Region Institute (Quzhou), University of Electronic Science and Technology of China, Quzhou, Zhejiang 324003 China; 3 School of Intelligence Science and Technology, Beijing University of Civil Engineering and Architecture, Beijing, 102616 China; 4 Faculty of Computing, Harbin Institute of Technology, Harbin, Heilongjiang 150001 China

**Keywords:** CircRNA–drug association prediction, Graph representation learning, Heterogeneous biological networks, Cross-modal feature fusion, Attention mechanism, Collaborative feature mining

## Abstract

**Background:**

Circular RNAs (circRNAs) are an emerging class of non-coding RNAs with covalently closed loop structures and have been increasingly recognized for their regulatory roles in disease progression and drug response. Accurately identifying circRNA–drug sensitivity associations is therefore essential for understanding therapeutic mechanisms and advancing precision medicine. However, most existing computational methods fail to effectively integrate semantic and structural information and overlook cross-modal feature co-optimization, thereby limiting their predictive performance.

**Results:**

To address these limitations, we develop an end-to-end graph representation learning framework for circRNA–drug sensitivity prediction by jointly modeling homogeneous similarity structures and heterogeneous interaction relationships. The framework integrates fused similarity graphs, semantic feature encoding with pre-norm residual attention, and structural representation learning via graph convolutional networks with Top-K sparse adjacency. In addition, a large-scale heterogeneous graph and a cross-modal collaborative feature mining module are employed to jointly optimize multi-source representations. Experimental results from 5-fold and 10-fold cross-validation, independent test evaluations, ablation study, and case study demonstrate that the proposed framework consistently achieves superior performance compared with state-of-the-art methods.

**Conclusions:**

The proposed framework provides a robust and effective computational strategy for circRNA–drug sensitivity prediction and offers a valuable tool for uncovering potential therapeutic associations, thereby facilitating future research in drug response analysis and precision medicine.

## Background

In recent years, circular RNAs (circRNAs) have attracted widespread attention due to their covalently closed loop structure and diverse regulatory functions in gene expression [1, 2]. Previous studies have demonstrated that circRNAs play important roles in regulating cellular processes such as proliferation, differentiation, apoptosis, and immune responses [3–5], and their aberrant expression is closely associated with various human complex diseases. For instance, some circRNAs act as competing endogenous RNAs (ceRNAs) by interacting with miRNAs to regulate transcriptional and translational levels of downstream genes, while others bind to RNA-binding proteins (RBPs) [6], thereby participating in post-transcriptional modifications and signaling pathway regulation [7]. These molecular mechanisms not only reveal the functional diversity of circRNAs but also provide important biological foundations for the development of computational prediction models [8–11].

Among existing methods, circRNA–RBP prediction models have been developed to identify potential circRNA–protein binding sites [12–14], aiding in the exploration of transcriptional regulation. CircRNA–disease prediction approaches, leveraging network representation learning and machine learning algorithms [15–20], have uncovered potential links between circRNA dysregulation and complex diseases such as cancer and neurodegenerative disorders. Additionally, circRNA–miRNA prediction tools, based on sequence matching, energy models, or deep learning frameworks [21–24], have facilitated the identification of circRNA–miRNA interactions and provided insights into the complexity of ceRNA regulatory networks [25]. These computational advances have offered a more systematic understanding of circRNA functions within molecular networks. Meanwhile, growing evidence suggests that circRNAs also play crucial roles in tumor chemoresistance and drug response mechanisms [26], with some circRNAs being closely associated with cellular sensitivity to specific chemotherapeutic agents [27]. This highlights the clinical potential of circRNAs in personalized treatment and targeted drug development, and underscores the importance of constructing efficient computational models to accurately predict circRNA–drug sensitivity associations [28, 29].

Nevertheless, traditional biomedical experimental methods for validating circRNA–drug associations often suffer from significant limitations in terms of cost and efficiency. On the one hand, wet-lab experiments such as qRT-PCR or RNA pull-down require substantial human, material, and time resources. On the other hand, given the inherently high combinatorial complexity of the circRNA–drug association space, experimental approaches alone are insufficient to achieve large-scale and systematic association screening. This substantially limits the efficiency of novel mechanism discovery and drug development. Therefore, the development of reliable computational methods cannot only significantly reduce experimental costs [30–35] but also provide valuable candidate information for subsequent validation [36–38], thereby improving the overall efficiency and precision of biomedical research [39–42].

Benefiting from the continuous advancements in drug association prediction and molecular representation learning, a large number of advanced representation and modeling techniques have been developed, providing important references for characterizing complex biomolecular relationships [43]. For example, multi-view contrastive learning improves the model’s ability to discriminate complex interaction patterns by establishing consistency constraints across different feature spaces [44]. Meanwhile, approaches based on large language models can extract semantically rich molecular representations from biomedical knowledge graphs and integrate them with graph neural networks to achieve unified modeling of multi-source heterogeneous information [45, 46]. In addition, multimodal fusion strategies further integrate molecular structure, sequence, and semantic information, significantly enhancing representation capability and model interpretability [47, 48]. These studies provide important methodological insights for circRNA–drug association prediction tasks. In this context, a number of computational models have been proposed for circRNA–drug sensitivity prediction. For example, in 2022, Deng et al. introduced GATECDA [49], which integrates circRNA sequence information, drug structural information, and known circRNA–drug sensitivity associations to compute circRNA–circRNA and drug–drug similarity matrices. They employed a graph attention autoencoder to learn low-dimensional embeddings, followed by a fully connected layer for prediction, achieving good performance in alleviating feature sparsity. However, this method relies solely on homogeneous similarity networks and struggles to capture cross-modal interactions, limiting predictive performance.

In 2023, Yang and Chen proposed MNGACDA [50], which constructs a multimodal network and employs a node-level graph attention autoencoder with residual modules to enhance feature extraction, followed by CNN-based multi-layer representation fusion. While predictive performance was improved, the model primarily focuses on local neighborhood similarity learning and overlooks potential global topological information.

In the same year, Li et al. developed the MNCLCDA framework [51], which applies random walk with restart (RWR) to preprocess similarity networks and mitigate noise. The model employs a hybrid neighborhood graph convolution network to extract multi-order neighborhood features, enhanced by graph-based contrastive learning to improve embedding discriminability and robustness. Prediction is then performed via dual-Laplacian regularized least squares. Although this approach balances local and global information to some extent, its ability to capture topological dependencies remains insufficient, and cross-modal semantic integration is still limited.

In 2024, Li et al. proposed DGATCCDA [52], which combines DeepWalk with graph attention networks to integrate global random walk information and local neighborhood features. Hierarchical attention and CNN modules were further employed for feature fusion, improving performance. However, this method is heavily dependent on DeepWalk-based pretraining, which may lead to insufficient information extraction in sparse networks.

Later in 2024, Xia et al. introduced SGTCDA [53], which integrates structural deep network embedding with graph Transformer models. This framework captures long-range dependencies and incorporates EdgeSHAPer to improve interpretability, while also emphasizing independent test set evaluation to avoid overly optimistic bias from cross-validation. Nonetheless, the model suffers from a decoupling between feature extraction and downstream modeling, lacking end-to-end optimization and deeper interaction feature mining.

In 2025, Liu et al. further developed MHGTCDA [54], incorporating stochastic autoencoders to enhance embedding diversity and robustness, and utilizing multi-layer heterogeneous graph Transformers to capture cross-modal interactions between circRNAs and drugs. Meanwhile, Niu et al. proposed MiGNN2CDS [29], which explores higher-order semantic relationship modeling by introducing a multiple instance learning mechanism. Specifically, pseudo meta-path instances are constructed in heterogeneous networks to capture potential association patterns between circRNA–drug pairs, and bidirectional embedding projection together with multi-scale attention mechanisms are employed to enhance representation capability and model interpretability. Although these methods have achieved certain performance improvements, they still exhibit limitations in cross-modal semantic alignment and the collaborative modeling of global and local information.

Overall, while existing approaches have advanced the field of circRNA–drug sensitivity prediction, several limitations persist: (i) feature extraction and downstream modeling are decoupled, hindering end-to-end optimization; (ii) structural information is underutilized, with insufficient integration of global topological and local structural features; and (iii) heterogeneous graph fusion remains limited, with inadequate modeling of semantic and deep cross-modal interactions.

To overcome these challenges, we propose AGCECDA (Attention-Guided Cooperative Embedding for CircRNA–Drug Association prediction), a novel end-to-end graph representation learning framework that jointly leverages homogeneous structural information and heterogeneous interaction patterns. Specifically, we construct a structure-aware similarity graph by integrating Gaussian Interaction Profile (GIP) kernels with multi-source similarity matrices, thereby obtaining high-quality circRNA and drug similarity representations. A dual-branch feature modeling strategy is then employed: one branch introduces a pre-norm residual attention mechanism to encode similarity graphs and leverages a Top-K sparse graph convolutional network to capture local structural patterns; the other branch constructs a heterogeneous interaction graph from known circRNA–drug associations to model high-order cross-modal dependencies. To effectively integrate multi-source graph embeddings, we design a heterogeneous graph cooperative representation fusion module to achieve complementary information integration from multiple perspectives. Finally, association scores are predicted via an inner product decoder.

**Fig. 1:**
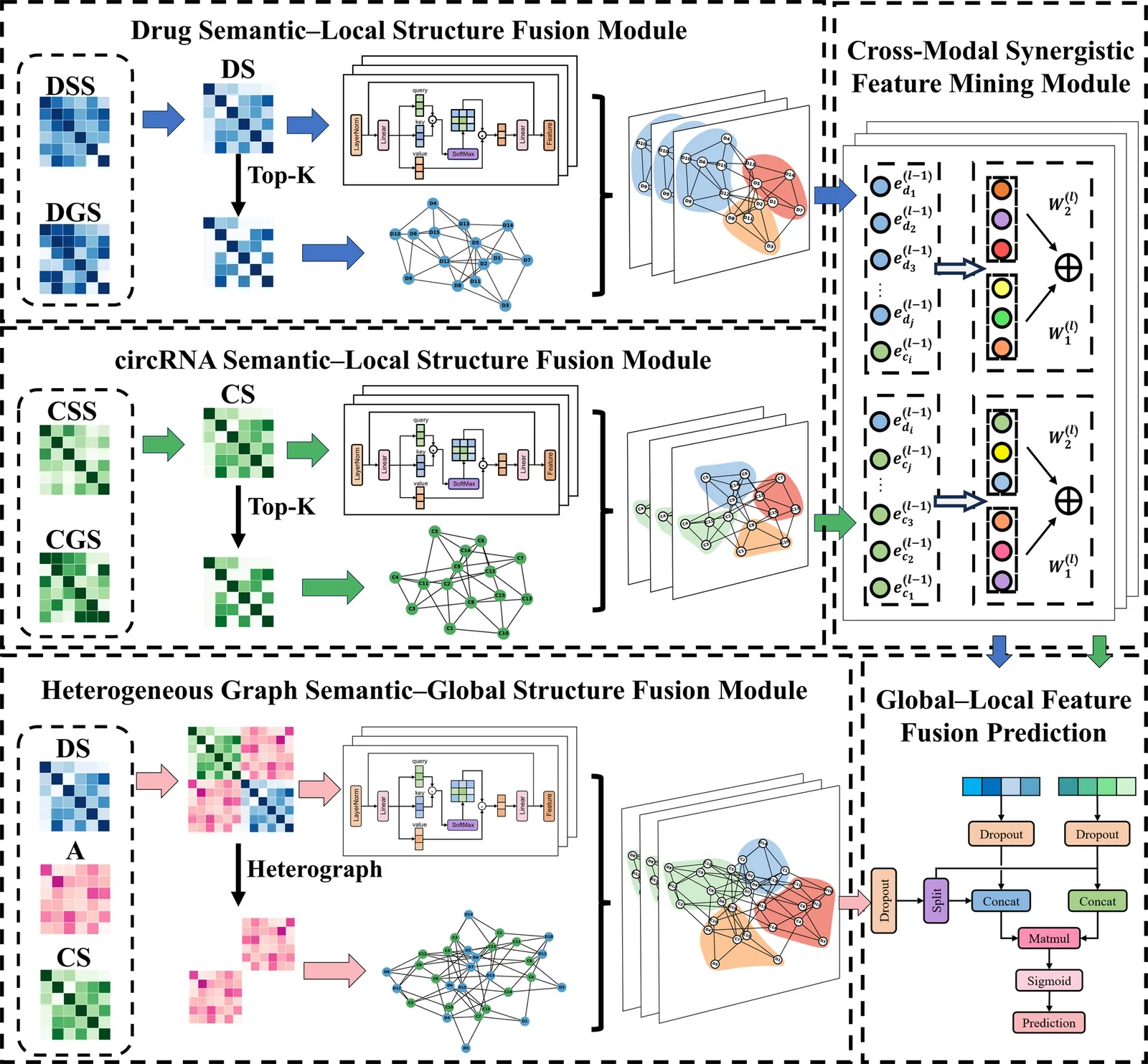
Architecture of AGCECDA

Extensive experiments, including 5-fold and 10-fold cross-validation, independent test evaluations, ablation study, and case study, demonstrate that AGCECDA outperforms state-of-the-art methods across multiple metrics such as AUC and AUPR, exhibiting superior generalization ability and robustness. Ablation studies further confirm the effectiveness of key components. Collectively, these results highlight the strong potential of AGCECDA for circRNA–drug sensitivity prediction and its promise in disease-related target identification and drug repositioning. The overall architecture of AGCECDA is illustrated in Fig. 1.

## Results and discussion

### Experimental settings and evaluation metrics

To comprehensively evaluate the performance of the proposed model, we adopted two commonly used validation strategies on benchmark datasets: 5-fold cross-validation (5-CV) and 10-fold cross-validation (10-CV). In the 5-CV experiments, we first randomly generated the same number of negative circRNA–drug sensitivity pairs as the verified positive samples, and then divided all samples into five equally sized subsets. Each subset was used as the test set in turn, while the remaining four subsets served as the training set. This process was repeated five times to ensure the reliability of the results. The 10-CV experiments followed a similar partitioning and validation strategy at a finer granularity.

Considering the potential issue of information leakage during cross-validation, which may lead to an overestimation of model performance, we further constructed an independent test set following the experimental design of SGTCDA. This independent set was used to evaluate the generalization ability and stability of the model on non-overlapping data.

For performance assessment, we employed seven widely used metrics in previous studies: the area under the receiver operating characteristic curve (AUROC), the area under the precision–recall curve (AUPR), accuracy, recall, precision, F1-score, and specificity. These metrics capture the classification ability of the model from different perspectives. Specifically, AUROC and AUPR measure the overall discriminative power, accuracy reflects overall prediction correctness, recall and precision emphasize sensitivity and exactness, F1-score balances recall and precision, while specificity evaluates the recognition ability for negative samples.

All experiments were conducted on Ubuntu 20.04 with an NVIDIA RTX 3090 GPU. The software environment included Python 3.7.4 and PyTorch 1.13.1 (CUDA 11.6). During model training, we used the AdamW optimizer with a learning rate of 1 × 10^−3^ and a dropout rate of 0.6. The number of attention heads was set to 2, and the number of k-nearest neighbors was set to 30 to achieve a balance between performance and stability.

### Performance comparison on cross-validation sets

**Fig. 2:**
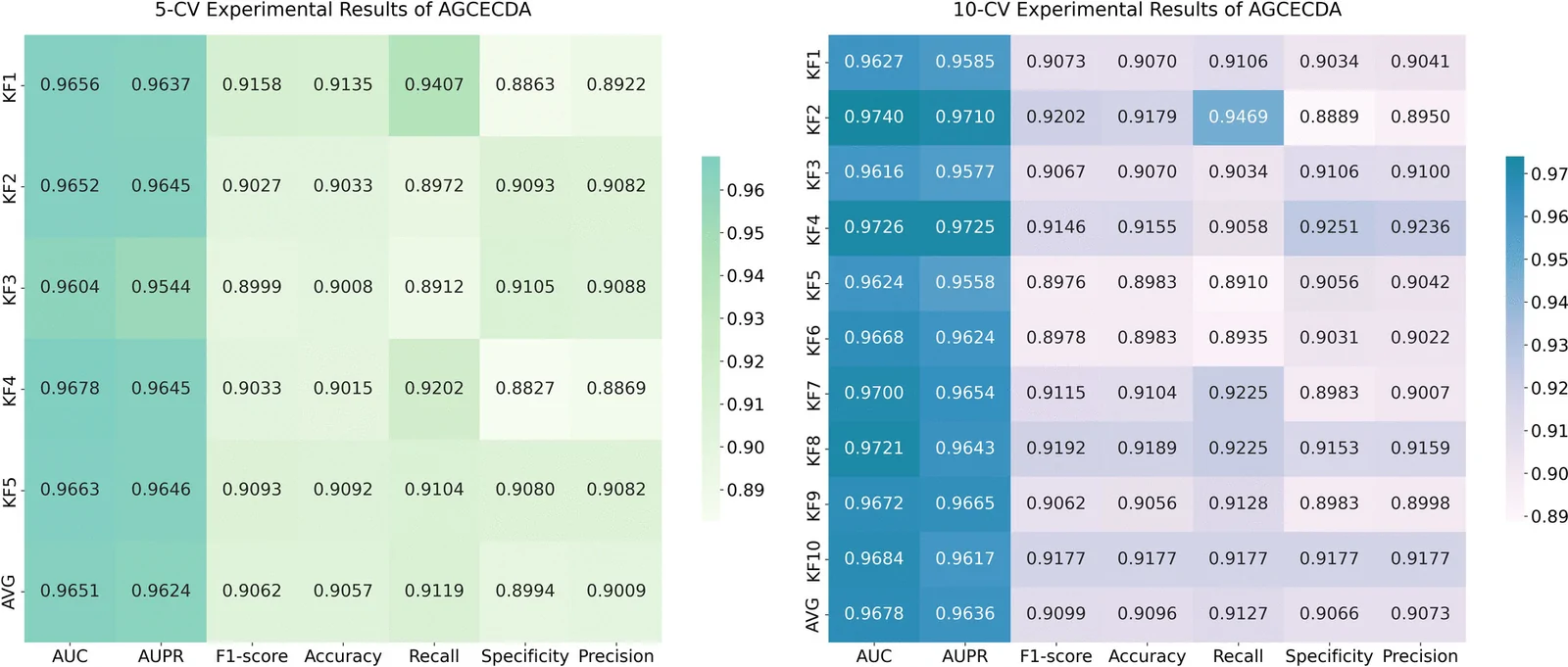
Performance heatmaps of AGCECDA under 5-fold and 10-fold cross-validation

**Fig. 3:**
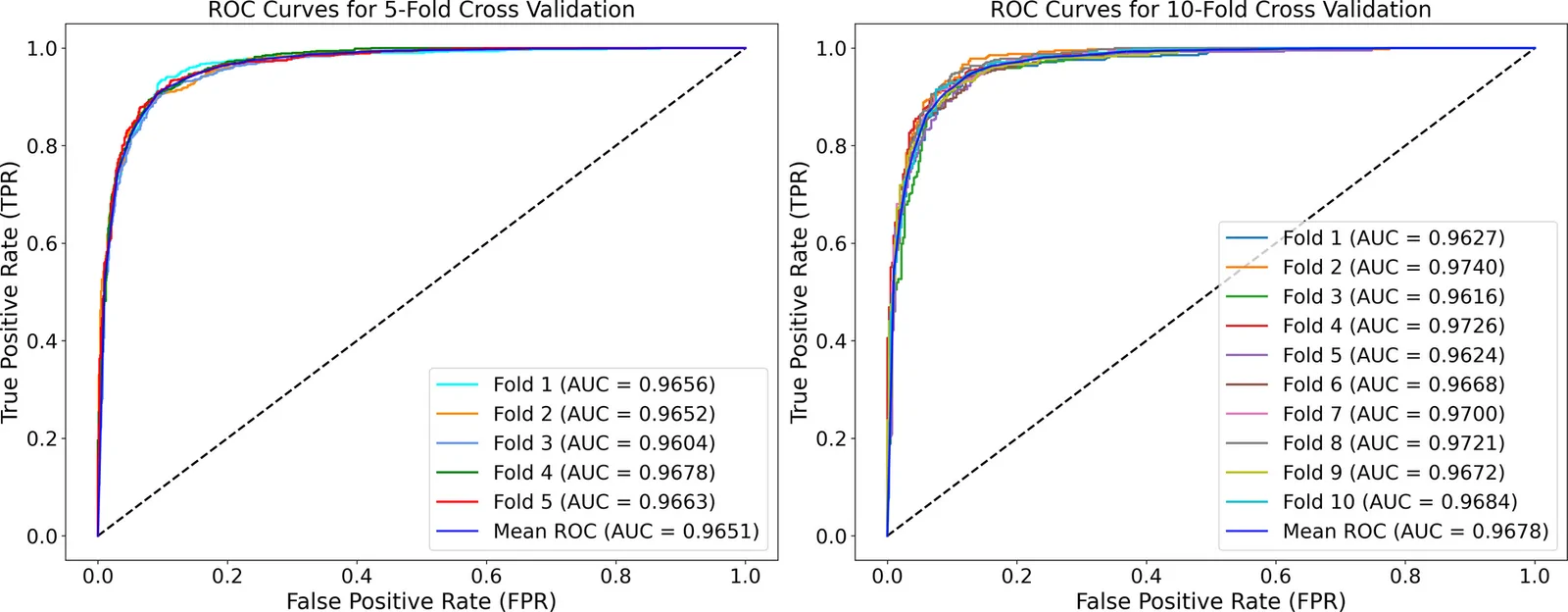
ROC curves of AGCECDA under 5-fold and 10-fold cross-validation

**Fig. 4:**
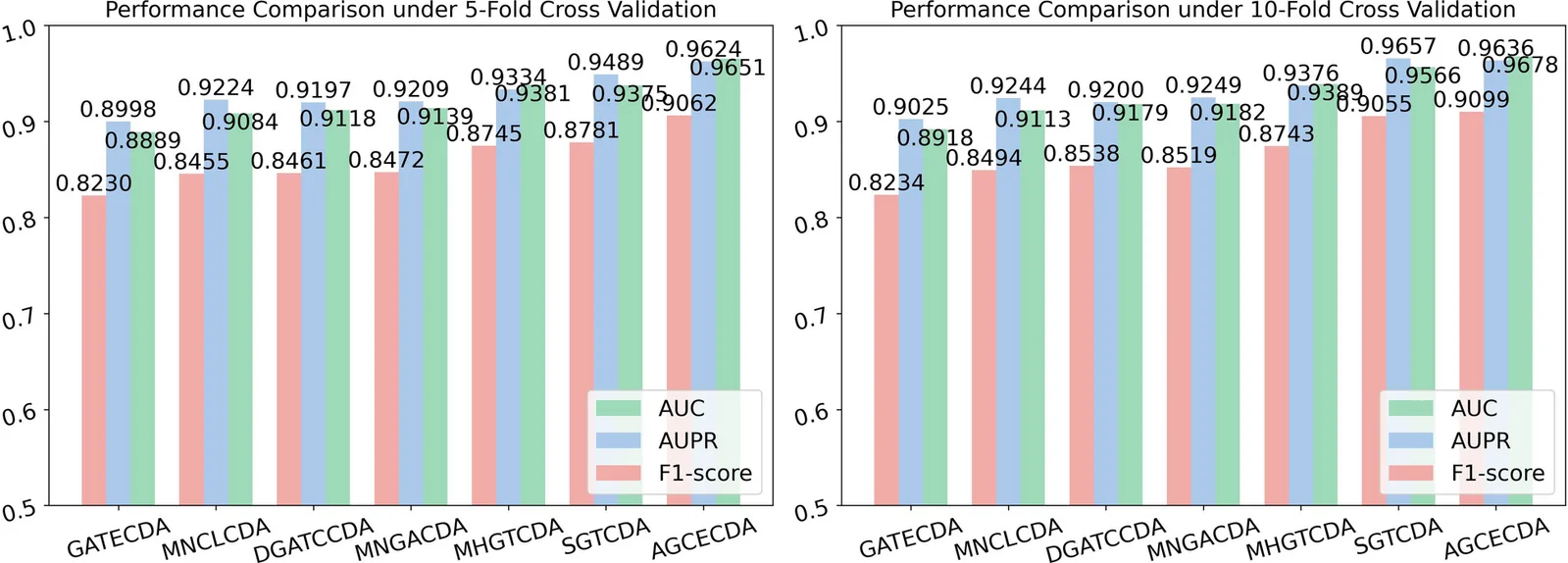
Performance comparison of AGCECDA and baseline methods under 5-fold and 10-fold cross-validation

To thoroughly evaluate the effectiveness of the proposed method in circRNA–drug sensitivity prediction, we performed both 5-CV and 10-CV experiments on a unified benchmark dataset, following the common practices in previous studies. Seven mainstream metrics were employed for systematic analysis, as illustrated in the performance heatmaps and ROC curves in Figs. 2 and 3. Moreover, the proposed AGCECDA method was compared with several state-of-the-art models, including GATECDA, MNCLCDA, DGATCCDA, MNGACDA, MHGTCDA, and SGTCDA, as shown in Fig. 4.

In the 5-fold cross-validation, AGCECDA exhibited consistently stable and superior performance across all metrics, with average AUC, AUPR, and F1-score reaching 0.9651, 0.9624, and 0.9062, respectively. The other metrics (Accuracy, Recall, Specificity, and Precision) also remained at high levels of around 0.90. As shown in the heatmap, the results across different folds (KF1–KF5) showed only minor fluctuations, while the ROC curves were smooth and closely approached the upper-left corner, indicating strong discriminative capability. Importantly, the bar chart comparison clearly demonstrates that AGCECDA outperformed SGTCDA and MHGTCDA in three key metrics: its AUC increased by 2.76% and 2.70%, its AUPR increased by 1.35% and 2.90%, and its F1-score increased by 2.81% and 3.17%, respectively. These findings confirm that AGCECDA provides significant advantages in overall discriminative power, the separation of positive and negative samples, and balanced prediction performance.

In the 10-fold cross-validation, the finer-grained data partitioning further confirmed the robustness of the proposed method. AGCECDA consistently maintained its leading performance across the three key metrics, achieving average AUC, AUPR, and F1-score values of 0.9678, 0.9636, and 0.9099, respectively. As shown in the heatmap, the results across the 10-folds (KF1–KF10) exhibited high consistency, and the ROC curves also demonstrated excellent classification performance. From the bar chart comparison, it is evident that AGCECDA achieved the best results in AUC and F1-score, surpassing SGTCDA and MHGTCDA by 1.12% and 2.89% in AUC, and by 0.44% and 3.56% in F1-score, respectively. Although AGCECDA achieved a slightly lower AUPR (0.9636) than SGTCDA (by 0.21%), it still outperformed MHGTCDA by 2.60%. This phenomenon may be attributed to the fact that AUPR is more sensitive to the coverage of positive samples in the predictions: in certain folds, SGTCDA demonstrated stronger performance in identifying a small number of high-confidence positive samples, thereby gaining a marginal advantage in AUPR. Nevertheless, the consistent superiority of AGCECDA in AUC and F1-score highlights its stronger robustness in overall discriminative capability and balanced prediction, enabling it to maintain superior performance under different partitioning conditions.

Overall, whether under 5-fold or 10-fold cross-validation, AGCECDA consistently outperformed the baseline methods across multiple metrics. This demonstrates that the proposed feature modeling and semantic–structural fusion strategy can achieve higher predictive accuracy and stronger robustness across different data partitioning scenarios.

### Performance comparison on the independent test set

Existing algorithms for predicting associations among circRNAs, drugs, and diseases have played an important role in promoting drug discovery, drug repositioning, and related studies. However, they generally suffer from several limitations. In cross-validation experiments, the entire association matrix is often used to construct similarity matrices or heterogeneous networks, which may lead to information leakage and cause the model’s performance on the validation set to be overestimated [53]. In addition, some methods fail to reinitialize the model object independently in each fold of *k*-fold cross-validation and instead reuse the same model instance, resulting in parameter sharing across folds. This implementation flaw indirectly exposes the entire dataset, thereby weakening the independence of the experiments and compromising the fairness of the results.

Based on this perspective, and to more objectively evaluate the model’s generalization capability and stability, we further adopted an independent test set following the experimental design of SGTCDA. In this setting, the training and test data are strictly separated, and the construction of similarity matrices and heterogeneous networks relies solely on association information within the training set, thus effectively avoiding bias introduced by validation-set leakage. It should be noted that, except for MHGTCDA, all comparative results were obtained from the SGTCDA paper, while the performance of MHGTCDA was reproduced by us using its released source code under the same data-splitting strategy.

**Fig. 5:**
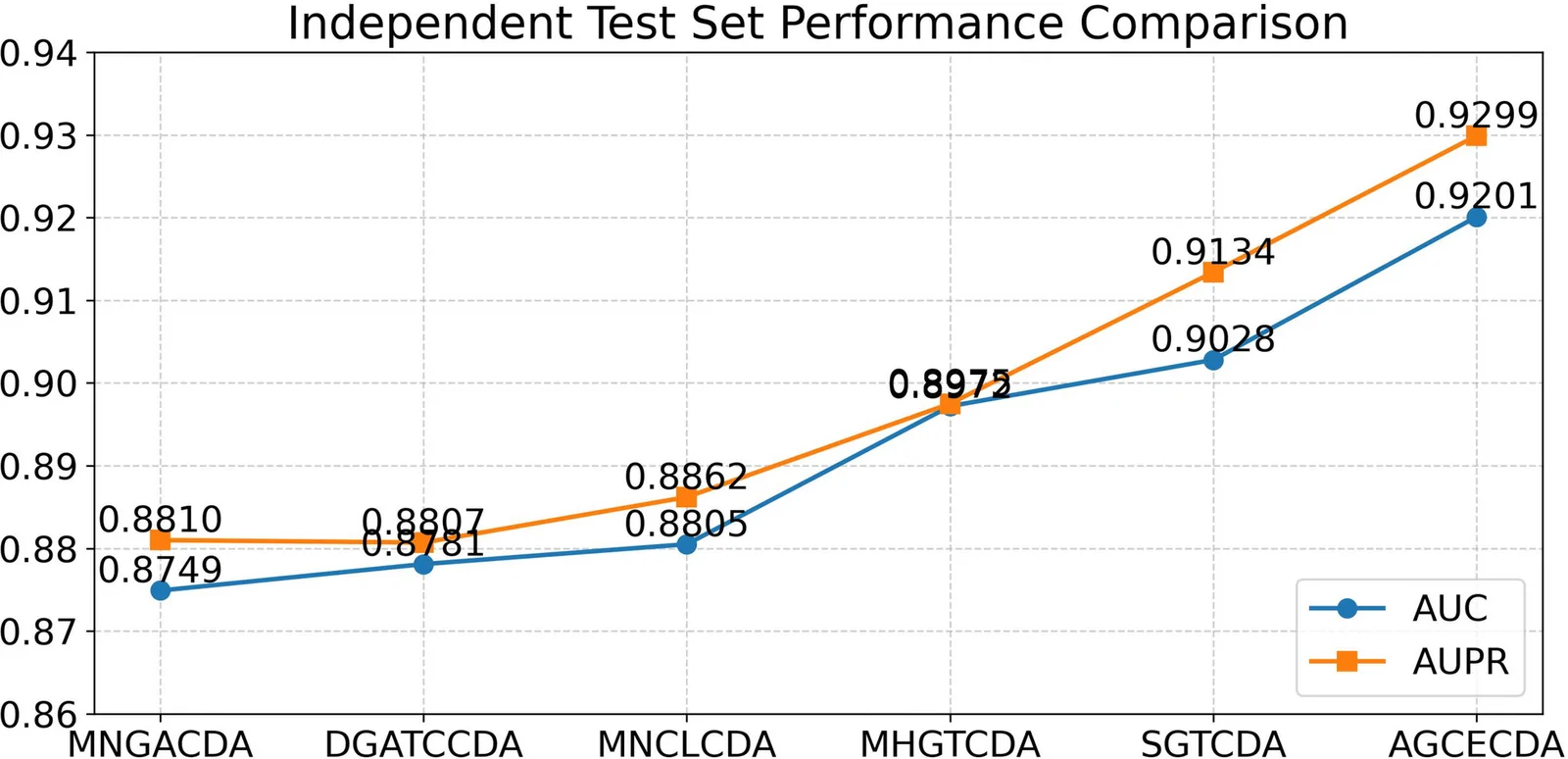
Visualization of algorithm performance comparison on the independent test set

The results on the independent test set demonstrate that AGCECDA exhibits stronger discriminative power and stability compared with several state-of-the-art models, as shown in Table 1 and Fig. 5. Specifically, AGCECDA achieved the best performance in three core metrics—AUC (0.9201), AUPR (0.9299), and F1-score (0.8483)—surpassing SGTCDA by 1.73%, 1.65%, and 0.44%, and outperforming MHGTCDA by 2.55%, 3.81%, and 3.92%, respectively, which is significantly better than other methods. Regarding auxiliary metrics, AGCECDA achieved an Accuracy of 0.8519, which is 1.42% higher than SGTCDA and 3.15% higher than MHGTCDA, further reflecting its stability in overall prediction.

**Table 1:** Performance comparison of different algorithms on the independent test set

Models	AUC	AUPR	F1-score	Accuracy	Recall	Specificity	Precision
MNGACDA	0.8749	0.881	0.8089	0.8019	0.8384	0.7654	0.7825
DGATCCDA	0.8781	0.8807	0.8147	0.8083	0.8424	0.7737	0.7892
MNCLCDA	0.8805	0.8862	0.8178	0.8115	0.846	0.7769	0.7919
MiGNN2CDS	0.8936	0.8963	0.8316	0.8235	0.8718	0.7751	0.7949
MHGTCDA	0.8972	0.8975	0.8163	0.8259	0.7739	0.8779	0.8637
SGTCDA	0.9028	0.9134	0.8439	0.8377	0.8536	0.8319	0.8345
AGCECDA	0.9201	0.9299	0.8483	0.8519	0.8283	0.8755	0.8693

**Fig. 6:**
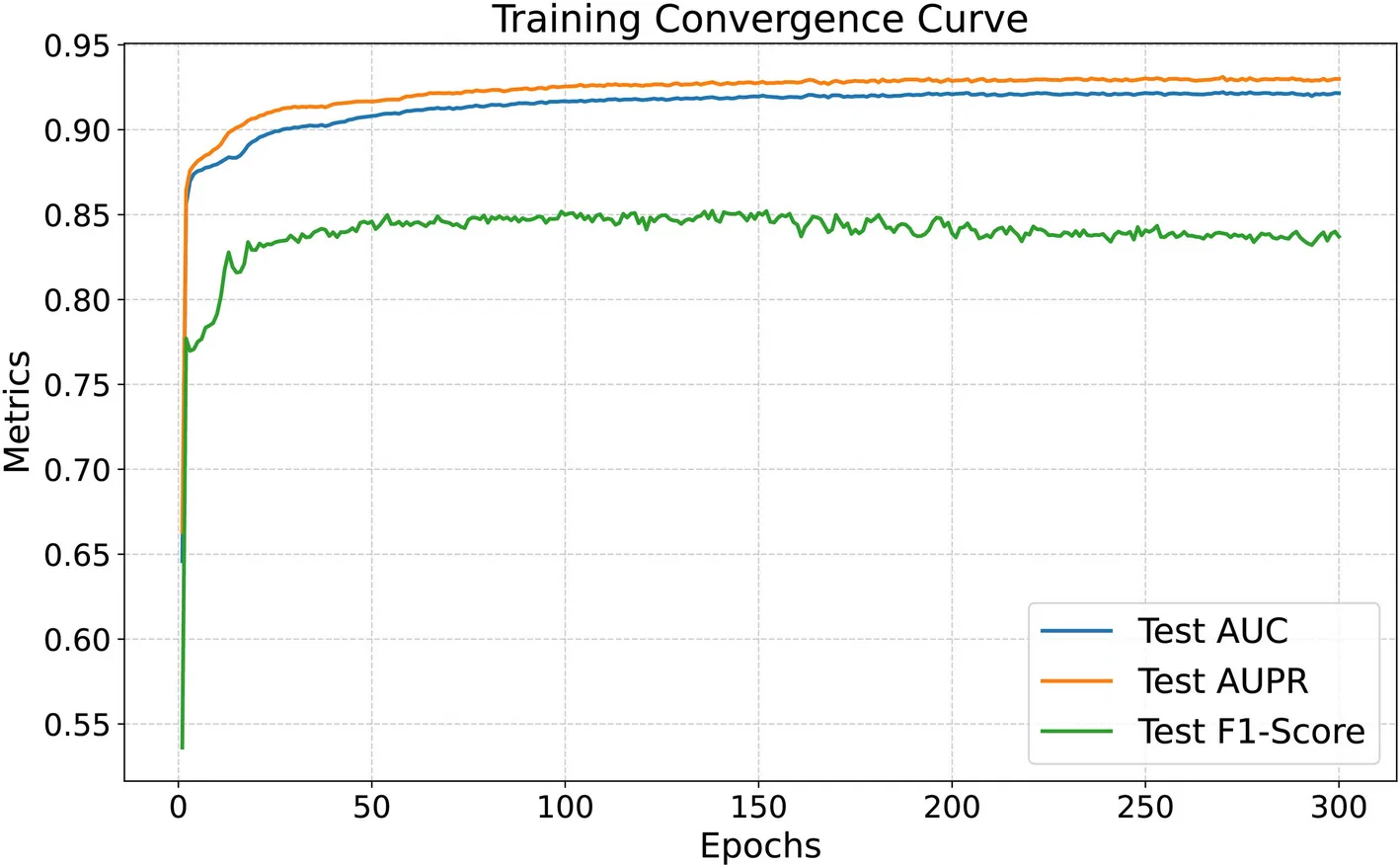
Convergence curves of different algorithms on the independent test set

It is noteworthy that, in terms of Recall, AGCECDA (0.8283) was lower than SGTCDA (0.8536). However, in Precision, AGCECDA (0.8693) was substantially higher than SGTCDA (0.8345, + 4.17%) and MHGTCDA (0.8637, + 0.65%). This indicates that while maintaining superior overall performance, AGCECDA places greater emphasis on reducing false positives and improving the reliability of positive sample predictions. In contrast, SGTCDA achieved slightly higher Recall but suffered from relatively lower Precision, suggesting an imbalance in its predictions. In addition, we plotted the convergence curves of the model (see Fig. 6), illustrating the variation trends of test AUC, AUPR, and F1-score with respect to training epochs. The results show that the model performance improves rapidly in the early stages of training and gradually stabilizes after approximately 50 epochs (out of a total of 300 epochs), indicating that the model can converge effectively and that the training process is stable, allowing training to be completed within an acceptable time cost.

In summary, the results on the independent test set clearly demonstrate that AGCECDA possesses stronger generalizability and robustness in realistic scenarios, consistently maintaining a comprehensive advantage over state-of-the-art baselines.

### Hyperparameter analysis

**Fig. 7:**
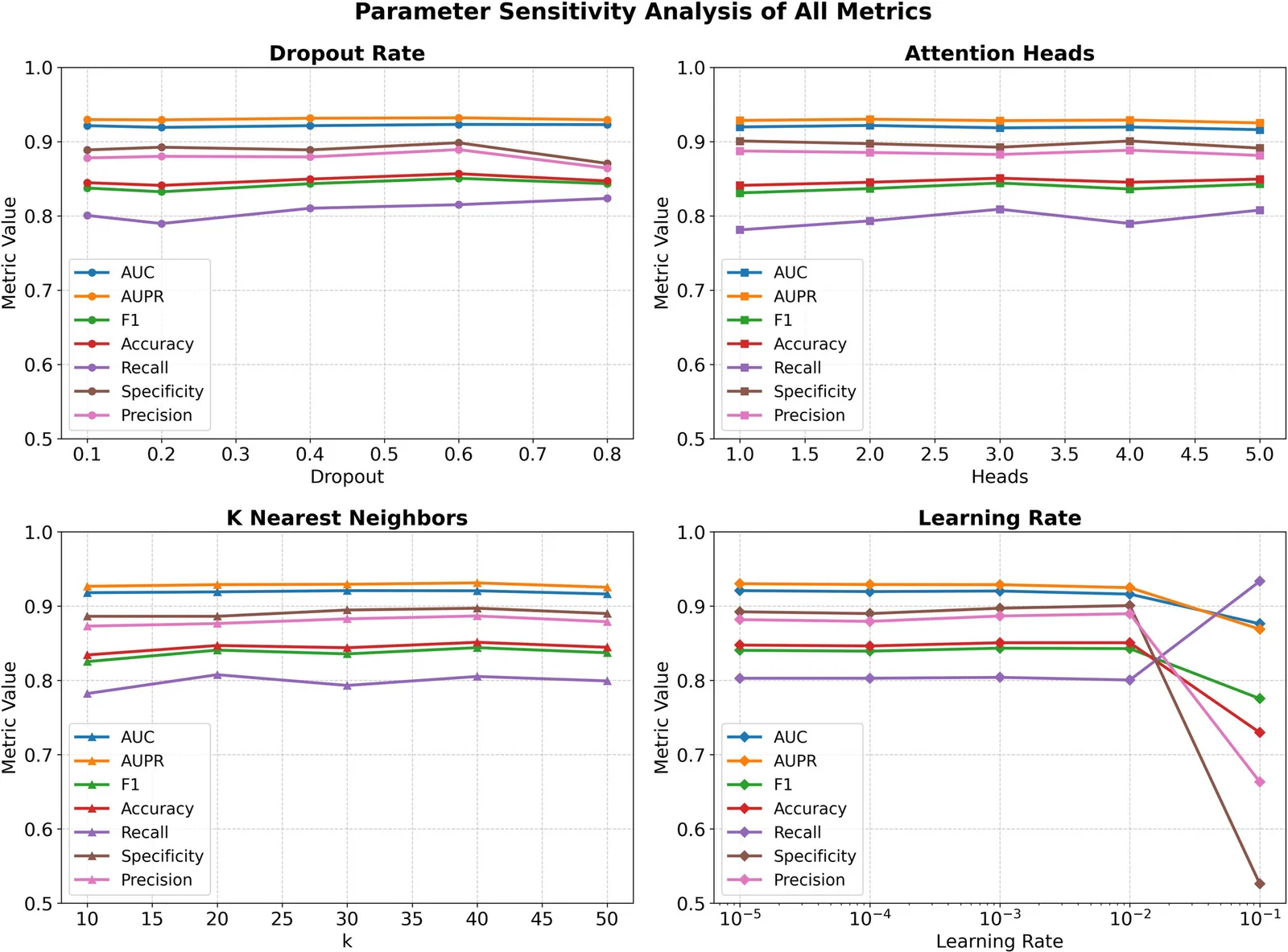
Hyperparameter sensitivity analysis results on the independent test set

To systematically evaluate the sensitivity of the model to key hyperparameters, we conducted a series of analyses on the independent test set, including the dropout rate, number of heads, *K*-nearest neighbors, and learning rate. The experimental results are shown in Fig. 7.

First, regarding the dropout rate, the model exhibits relatively stable performance within the range of 0.1 to 0.8. Among them, when the dropout is set to 0.6, all evaluation metrics (e.g., AUC, AUPR, F1-score, and accuracy) reach relatively optimal values. This indicates that an appropriate dropout mechanism helps alleviate overfitting and improve the generalization ability of the model, while excessively low or high dropout rates may limit the model’s expressive capacity or introduce excessive information loss.

Second, for the number of attention heads, the experimental results show that the model performs best when the number of heads is between 2 and 3. This suggests that the multi-head attention mechanism can capture feature information from different subspaces; however, too many heads may introduce redundant information and increase model complexity, thereby slightly degrading performance.

In the analysis of the *K*-nearest neighbors parameter, it can be observed that the model remains relatively stable when *K* ranges from 20 to 40. This implies that an appropriately enlarged neighborhood range helps capture richer structural information, whereas excessively large *K* values may introduce noisy neighbors, negatively affecting model performance.

Finally, regarding the learning rate, the model demonstrates stable and strong performance when the learning rate is within the range of 1e − 5 to 1e − 3. However, when the learning rate increases to 0.1, a significant performance degradation is observed, particularly in metrics such as Accuracy, Precision, and Specificity. This indicates that an excessively large learning rate may lead to unstable training or even failure to converge.

Overall, AGCECDA demonstrates strong robustness within reasonable hyperparameter ranges, with relatively stable variations across all performance metrics, indicating that the model is not highly sensitive to hyperparameters and possesses good stability and generalization capability. Based on the above analysis, the optimal parameter configuration is selected as follows: dropout = 0.6, number of heads = 2, *K*-nearest neighbors = 30, and learning rate = 1e − 3.

### Ablation study

**Fig. 8:**
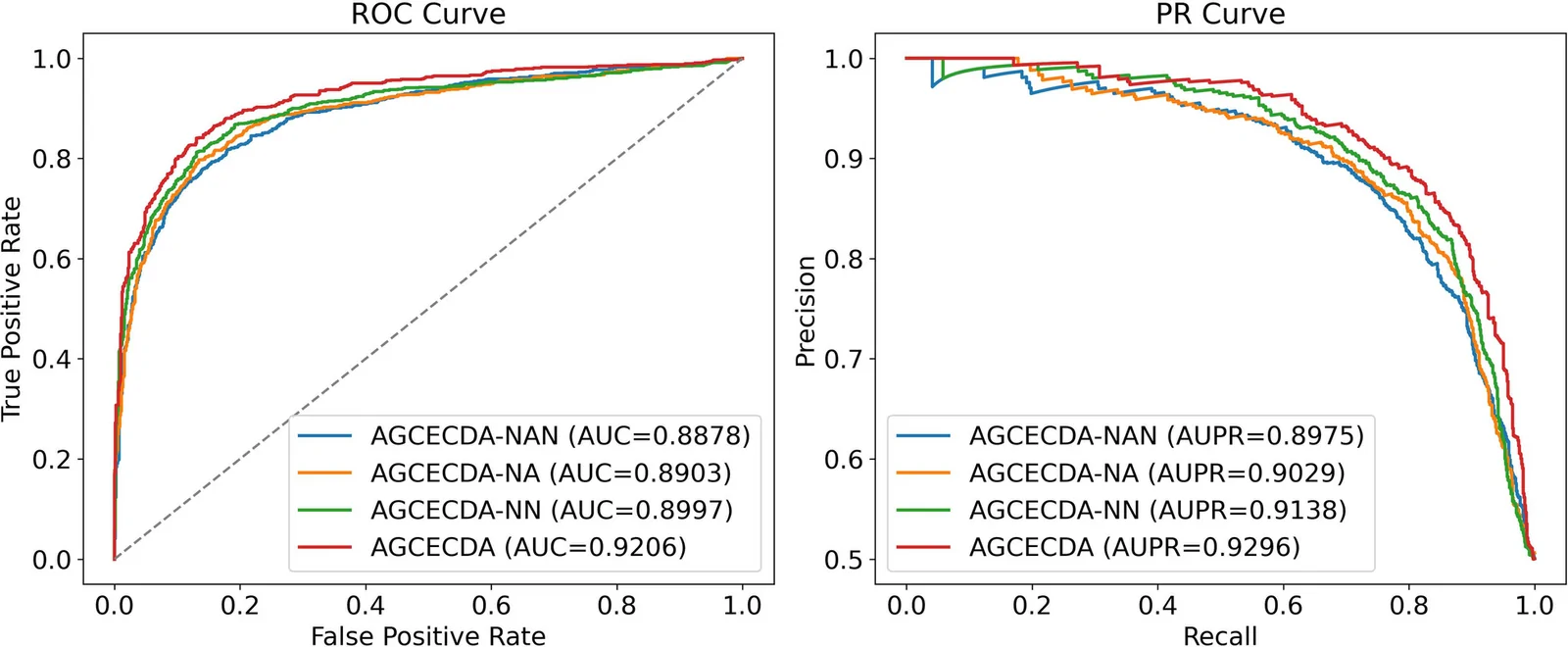
Visualization of AGCECDA ablation study results on the independent test set

To verify the effectiveness of each core component in AGCECDA, we designed three variants based on the full model: (i) no_attention (NA), which removes the pre-norm residual attention mechanism; (ii) no_NGCF (NN), which excludes the cross-modal collaborative feature mining module; and (iii) no_attention_and_NGCF (NAN), which simultaneously removes both modules. We then conducted ablation experiments on the independent test set, comparing the performance of the full model with these variants, as summarized in Table 2 and shown in Fig. 8.

**Table 2:** Ablation study results of AGCECDA on the independent test set

Models	AUC	AUPR	F1	Accuracy	Recall	Specificity	Precision
AGCECDA-NAN	0.8878	0.8976	0.8111	0.8186	0.7787	0.8585	0.8463
AGCECDA-NA	0.8903	0.903	0.8241	0.8289	0.8017	0.8561	0.8478
AGCECDA-NN	0.8997	0.9139	0.8253	0.8331	0.7884	0.8779	0.8659
AGCECDA	0.9206	0.9297	0.8477	0.8525	0.821	0.8839	0.8761

When both the pre-norm residual attention and the cross-modal collaborative feature mining module were removed (NAN), the model’s performance dropped substantially, with AUC and AUPR reduced to 0.8878 and 0.8976, and F1 falling to 0.8111. This result highlights that semantic enhancement and cross-modal feature fusion play complementary roles in improving predictive discriminability, and both are indispensable.

After removing only the attention mechanism (NA), the model achieved AUC and AUPR values of 0.8903 and 0.903, showing slight improvement compared to NAN. This indicates that even without semantic attention, cross-modal feature fusion can still provide benefits. However, compared with the full model (AUC = 0.9206, AUPR = 0.9297), the gap remains significant, underscoring the importance of semantic attention in alleviating feature compression and maintaining diversity in information representation.

Further, when the cross-modal collaborative feature mining module was removed (NN), the model achieved AUC and AUPR values of 0.8997 and 0.9139, which overall outperformed the no_attention variant. This suggests that the attention mechanism makes a stronger contribution to enhancing semantic feature modeling, while the absence of cross-modal collaboration weakens the joint modeling of global semantics and local structures.

Overall, the complete AGCECDA model achieved the best performance across key metrics (AUC = 0.9206, AUPR = 0.9297, F1 = 0.8477), significantly outperforming all ablated variants. These results fully demonstrate that both the Pre-Norm Residual Attention mechanism and the cross-modal collaborative feature mining module are indispensable. Their synergy effectively alleviates feature over-compression and structural information loss in existing methods, thereby enabling AGCECDA to achieve superior performance in circRNA–drug sensitivity prediction.

## Case study

To further validate the reliability and practical value of the model in predicting circRNA–drug sensitivity associations, we conducted a case study using the independent CTRP database [55], employing drug response difference (DRD) and false discovery rate (FDR) as significance metrics (associations with FDR < 0.05 were considered significant). The results demonstrated that multiple predicted associations were supported by CTRP, as summarized in Table 3, confirming the model’s capability to capture potential biological relevance.

**Table 3:** Top 20 predicted circRNA–drug associations with validation

Drug_name	CircRNA_name	DRD	FDR	Evidence	Association
Vorinostat	ANP32B	− 0.2841	6.57E − 03	CTRP	True
SNX-2112	EFEMP1	1.2517	5.29E − 06	CTRP	True
Docetaxel	PTMS	1.6119	6.09E − 03	CTRP	True
Linifanib	CRIM1	0.6972	1.63E − 11	CTRP	True
Foretinib	ANP32B	− 0.3206	4.68E − 02	CTRP	True
Axitinib	POLR2A	− 0.6061	1.13E − 03	CTRP	True
Belinostat	PLAU				Unconfirmed
Methotrexate	MEF2D	− 1.0417	3.03E − 03	CTRP	True
PHA-793887	RPS6	− 0.4593	7.51E − 02	CTRP	False
OSI-930	CTSB	0.3440	7.41E − 02	CTRP	False
Sunitinib	KRT19	0.6229	1.80E − 10	CTRP	True
AZD7762	ADGRG1	0.7457	2.93E − 03	CTRP	True
AZD8055	ASPH	0.8475	1.82E − 05	CTRP	True
Pazopanib	SPARC	0.3332	4.40E − 02	CTRP	True
PI-103	ESRP2				Unconfirmed
piperlongumine	FBLN1	0.3897	5.18E − 04	CTRP	True
Afatinib	ANP32B				Unconfirmed
Bexarotene	PEA15	0.1106	1.87E − 01	CTRP	False
Imatinib	FBLN1	0.4166	2.42E − 02	CTRP	True
Masitinib	KRT7	0.3590	2.66E − 02	CTRP	True

A typical example is the association between Linifanib and circRNA CRIM1, which was the most significant (FDR = 1.63E − 11). Linifanib is an oral small-molecule tyrosine kinase inhibitor primarily targeting VEGFR and PDGFR, commonly used to inhibit angiogenesis and tumor cell proliferation [56]. The model’s prediction aligns well with its known anti-angiogenic mechanism, suggesting that CRIM1 may play a critical regulatory role in angiogenesis-related pathways, thereby influencing Linifanib sensitivity [57]. Another representative case is the significant association between Sunitinib and KRT19 (FDR = 1.80E − 10). Sunitinib is a multi-targeted tyrosine kinase inhibitor widely used for renal cell carcinoma and gastrointestinal stromal tumors [58]. Previous studies have indicated that KRT19 can serve as a prognostic and drug sensitivity biomarker in various cancers [59]. Our results further support the potential relationship between this molecule and targeted drugs, providing new insights for the development of combined biomarkers in clinical settings.

Additionally, the association between Piperlongumine and FBLN1 was also validated in CTRP (FDR = 5.18E − 04). Piperlongumine is a natural compound with multiple pharmacological activities, particularly showing unique anticancer properties [60]. FBLN1, as an extracellular matrix-related gene, may influence tumor cell invasion and metastasis when aberrantly expressed [61]. The predicted association highlights a novel mechanism, suggesting that circRNAs also play a key role in modulating natural drug sensitivity.

Moreover, the model predicted and validated several other novel associations, including docetaxel–PTMS [62], methotrexate–MEF2D [63], axitinib–POLR2A [64], and AZD8055–ASPH [65], further expanding the drug–circRNA interaction network. It should be noted that some predicted associations were not verified in CTRP, which may represent genuine novel relationships or reflect database coverage limitations, warranting further experimental validation. Overall, the case study indicates that AGCECDA can identify biologically and clinically meaningful associations, providing new directions for biomarker discovery and personalized therapy.

## Conclusions

In this study, we addressed the limitations of existing circRNA–drug sensitivity prediction methods, including insufficient semantic–structural feature fusion and the lack of cross-modal feature collaborative optimization, by proposing an end-to-end graph representation learning framework, AGCECDA. This approach integrates similarity graph construction, Pre-Norm Residual Attention, local–global structural graph convolution embeddings, and heterogeneous graph modeling to achieve multi-level collaborative representation of semantic and structural features. On this basis, a cross-modal collaborative feature mining module was further designed to effectively integrate multi-source graph embeddings, enabling the model to comprehensively capture the potential global–local interaction patterns between circRNAs and drugs. Across multiple experiments, AGCECDA consistently outperformed existing methods in 5-fold and 10-fold cross-validation as well as on an independent test set, demonstrating both stability and strong generalization capability. Ablation studies confirmed that cross-modal collaborative feature mining and global–local structural fusion are critical for performance enhancement, while the case study further validated its potential to uncover novel circRNA–drug sensitivity associations. In summary, AGCECDA provides an efficient and robust solution for circRNA–drug sensitivity prediction and offers strong support for biomarker discovery and personalized treatment strategies in precision medicine.

## Methods

### Data preparation and preprocessing

In this study, we adopted the benchmark dataset curated by Deng et al. [49] as the experimental data source. This dataset has been widely recognized as a standard benchmark for algorithmic performance comparison in this field [50–54]. Specifically, the circRNA–drug sensitivity associations were initially collected from the circRic database [66], which integrates circRNA expression profiles across multiple cancer cell lines and their regulatory roles in drug response. Drug sensitivity information was obtained from the GDSC database [67], which systematically records the responses of cancer cell lines to various anticancer agents along with related genomic features.

The original dataset contained 404 circRNAs and 250 drugs, corresponding to 80,076 potential associations. Subsequently, the influence of circRNAs on drug response was evaluated with reference to the Cancer Cell Line Encyclopedia (CCLE), and statistically significant associations were identified using the Wilcoxon rank-sum test. By controlling the false discovery rate (FDR < 0.05), a total of 4134 significant associations between 271 circRNAs and 218 drugs were finally obtained. Based on this, a bipartite association matrix $A\in {\mathbb{R}}^{271\times 218}$ was constructed, where ${A}_{ij}=1$ indicates a sensitivity association between circRNA i and drug j, and ${A}_{ij}=0$ otherwise. To further extract molecular-level feature information, we retrieved the host gene sequences of cirscRNAs from the NCBI Gene database [68] and calculated circRNA similarity based on the Levenshtein distance, resulting in the circRNA sequence similarity matrix (CSS). In parallel, chemical structure information of drugs was downloaded from PubChem [69], and drug–drug similarities were computed using the RDKit toolkit [70] and the Tanimoto method, yielding the drug structure similarity matrix (DSS).

### Calculation of GIP kernel similarity

To preserve more information in circRNA–drug similarity modeling, we incorporate the Gaussian Interaction Profile (GIP) kernel similarity on top of the existing similarity matrices. This method is based on the assumption that if two biomolecules (circRNAs or drugs) exhibit more similar interaction patterns with another type of objects, they are functionally more similar. Following this idea, we separately calculate the GIP kernel similarity matrices for circRNAs and drugs, which are subsequently used as essential inputs for downstream modeling.

For circRNAs, the GIP kernel similarity is defined as:

1 $$CGS = (c_i,c_j) = \mathrm{exp}\left(-\alpha_{c}\|\mathrm{G}(c_i)-G(c_j)\|^2\right)$$

where $G\left({c}_{i}\right)$ denotes the interaction feature vector of circRNA ${c}_{i}$​, and the scale parameter ${\alpha}_{c}$​ is defined as:

2 $$\alpha_c=\frac{1}{\frac{1}{n_c}\sum\limits_{i=1}^{n_c}\|\mathrm{G}(c_i)\|^2}$$

where ${n}_{c}$​ representing the number of circRNAs. Consequently, the circRNA GIP similarity matrix is obtained as $CGS\in {\mathbb{R}}^{{n}_{c}\times {n}_{c}}$.

Similarly, the GIP kernel similarity for drugs is formulated as:

3 $$DGS (d_i,d_j) = \mathrm{exp}(-\alpha_d\|\mathrm{G}(d_i)-\mathrm{G}(d_j)\|^2)$$

where the parameter ${\alpha}_{d}$​ is defined as:

4 $$\alpha_{d} = \frac{1}{\frac{1}{n_{d}} \sum\limits^{n_{d}}_{i=1} \|\mathrm{G} (d_{i})\|^{2}}$$

where ${n}_{d}$​ denoting the number of drugs. Thus, the drug GIP similarity matrix is constructed as $DGS\in {\mathbb{R}}^{{n}_{d}\times {n}_{d}}$​.

### Similarity integration and heterogeneous graph construction

Relying solely on a single source of similarity is insufficient to comprehensively characterize the relationships among biomolecules. Therefore, we further integrate the existing similarities with the Gaussian Interaction Profile (GIP) similarities. Specifically, the integrated similarity for circRNAs (CS) is defined as:

5 $$CS_{ij} \left\{\begin{array}{ll} \frac{CSS_{ij} + CGS_{ij}}{2\vphantom{\frac{1}{2}}}, & \mathrm{if}\ CSS_{ij} \neq 0,\\ CGS_{ij}, & \mathrm{otherwise} \end{array} \right.$$

where CSS denotes the circRNA sequence similarity matrix.

Similarly, the integrated similarity for drugs (DS) is given as:

6 $$DS_{ij} \left\{\begin{array}{ll} \frac{DSS_{ij} + DGS_{ij}}{2\vphantom{\frac{1}{2}}}, & \mathrm{if}\ DSS_{ij} \neq 0,\\ DGS_{ij}, & \mathrm{otherwise} \end{array} \right.$$

where DSS represents the drug chemical structure similarity matrix.

In the process of feature modeling, effectively integrating the multi-source relationships between circRNAs and drugs is a critical step. To this end, based on the homogeneous similarity structures, we incorporate circRNA–drug interaction information to construct a large-scale heterogeneous graph consisting of multiple types of nodes and edges. Concretely, we jointly encode the circRNA similarity matrix *CS*, the drug similarity matrix *DS*, and the circRNA–drug association matrix *A*, resulting in the following overall representation:

7 $$H = \left[\begin{array}{cc} CS & A^{T}\\ A & DS \end{array}\right]$$

This matrix not only preserves the structural similarities among circRNAs and among drugs but also explicitly models the cross-modal circRNA–drug interactions. In this way, both homogeneous and heterogeneous information are integrated into a unified graph representation framework. Such a construction provides a solid foundation for subsequent semantic and structural fusion learning, enabling the model to capture richer cross-domain dependency patterns.

### Semantic and structural feature modeling

Conventional approaches predominantly rely on graph neural networks (GNNs) to model features within circRNA–drug networks, typically propagating information through adjacency or similarity matrices. However, during multi-layer iterative aggregation, GNNs are prone to the issue of neighbor feature homogenization (over-smoothing), whereby node representations gradually converge and obscure the underlying heterogeneity and higher-order semantic relationships. This limitation is particularly pronounced in circRNA–drug prediction tasks, where circRNAs and drugs exhibit highly complex and nonlinear interactions in both semantic and structural spaces. Relying solely on a single graph propagation mechanism is often insufficient to capture such multi-level information, resulting in the model’s inability to effectively distinguish different types of interaction features.

In contrast, we propose a semantic–structural fusion feature modeling strategy, which introduces complementary semantic information while maintaining graph-structured representations, thereby alleviating the information loss caused by single-path GNN aggregation. Through this integration, the model not only preserves the global relationships reflected by graph topology but also leverages semantic features to uncover deeper biological connections between circRNAs and drugs, ultimately enhancing predictive accuracy and generalization capability.

Specifically, at the semantic level, we model the circRNA integrated similarity matrix (*CS*), the drug integrated similarity matrix (*DS*), and the heterogeneous graph matrix (*H*) independently. Each matrix is encoded using the Pre-Norm Residual Attention mechanism [14]. This mechanism enables stable training while effectively capturing higher-order semantic dependencies across multi-source similarities, yielding the corresponding embedding representations. Formally, given an input matrix $X\in {\mathbb{R}}^{n\times n}$, where *n* denotes the number of nodes, a single-layer Pre-Norm Residual Attention operation can be defined as:

8 $$X_{norm} = \mathrm{LayerNorm}(\mathrm{MLP}(X))$$

9 $$X_{\mathrm{atten}} = \mathrm{Attention} (X_{norm}) = \mathrm{Softmax} \left( \frac{QK^{\top}}{\sqrt{d_{k}}} \right)V$$

10 $$\tilde{X} = X_{norm} + X_{\mathrm{atten}}$$

Here, LayerNorm denotes layer normalization; *Q*, *K*, and *V* correspond to the query, key, and value matrices in the attention mechanism; ${d}_{k}$ is the scaling factor used to prevent excessively large dot products; and $\widetilde{X}$ represents the output features after residual updating.

At the structural level, we first construct Top-*K* sparse adjacency matrices for the circRNA integrated similarity matrix (*CS*) and the drug integrated similarity matrix (*DS*). This operation retains only the most significant *K* neighbors for each node, thereby effectively mitigating noise introduced by spurious edges while avoiding redundancy caused by overly dense adjacency relationships. Consequently, this strategy emphasizes the most critical topological features.

For the heterogeneous graph *H*, we utilize only the graph association relation ${\widetilde{A}}_{h}$, namely the circRNA–drug interaction links, as the core connectivity pattern. This design ensures that the learning process focuses on the key cross-modal associations, while preventing potential structural bias or semantic noise that could be introduced by other homogeneous edges.

Based on these constructions, the graph convolutional network (GCN) propagation rule is defined as:

11 $$\tilde{A}_{c} (i, j) = \left\{ \begin{array}{ll} CS(i,j), & j \in \mathrm{TopK}_{i} (CS)\\ 0, & \mathrm{otherwise} \end{array}\right.$$

12 $$\tilde{A}_{d} (p, q) = \left\{ \begin{array}{ll} DS(p,q), & q \in \mathrm{TopK}_{p} (DS)\\ 0, & \mathrm{otherwise} \end{array}\right.$$

13 $$\tilde{A}_{h} = \left[ \begin{array}{cc} {} & A^{T}\\ A & {} \end{array} \right]$$

14 $$Z^{(l+1)} = \sigma (\tilde{D}^{-1/2}\tilde{A}\tilde{D}^{-1/2}{Z}^{(l)}W^{(l)})$$

where $\widetilde{A}$ is the normalized adjacency matrix, $\widetilde{D}$ is its degree matrix, $Z^{(l)}$ is the feature representation at the l th layer, ${W}^{(l)}$ is the trainable weight matrix, and $\sigma \left(\cdot \right)$ denotes the nonlinear activation function.

### Cross-modal feature mining

To further exploit multi-source graph embedding information, we design a cross-modal cooperative feature mining module [71]. In this module, information exchange and feature aggregation are performed across heterogeneous node types (e.g., circRNAs and drugs) through a message-passing mechanism, thereby enabling deep joint modeling of semantic and structural information.

From a theoretical perspective, this module follows the message passing framework in graph neural networks, where each node iteratively updates its representation by aggregating information from its neighbors. Unlike conventional homogeneous aggregation, our method explicitly introduces cross-modal interaction modeling, allowing semantic correlations between heterogeneous biological entities to be captured during propagation. Specifically, at each layer, the representation of a node depends not only on its historical embedding but also incorporates information from its cross-modal neighbors. This mechanism can be interpreted as a joint feature transformation process that integrates self-information and neighbor-induced semantic interaction features.

During the propagation process at the *l*th layer, the feature update of a circRNA node *c* can be formalized as:

15 $$\mathbf{h}^{(l+1)}_{c} = \sigma \left(\mathbf{W}^{(l)}_{1} \mathbf{h}^{(l)}_{c} + \sum\limits_{d \in \mathcal{N}_{c}} \alpha_{dc} \mathbf{W}^{(l)}_{2} \left(\mathbf{h}^{(l)}_{d} \oplus \left( \mathbf{h}^{(l)}_{d} \odot \mathbf{h}^{(l)}_{c} \right)\right)\right)$$

where ${h}_{c}^{(l)}$ denotes the embedding of circRNA at the *l*th layer, ${h}_{d}^{(l)}$ represents the embedding of its neighboring node (e.g., a drug), ⊕ indicates vector concatenation, ⊙ denotes the element-wise product, and ${\alpha}_{dc}$ is a symmetric normalization coefficient introduced to alleviate the bias caused by degree differences. $\sigma \left(\cdot \right)$ is a nonlinear activation function. The first term ${W}_{1}^{(l)}{h}_{c}^{(l)}$ denotes a linear transformation of the node’s own features, which preserves its historical semantic information. The second term corresponds to the aggregation of cross-modal neighbor information.

This propagation mechanism preserves the structural constraints while explicitly modeling semantic interactions between cross-modal nodes.

To enable batch propagation in a unified manner, we stack all node embeddings into a matrix ${E}^{(l)}$, and perform matrix-based operations to achieve efficient global feature updates across the entire heterogeneous graph:

16 $$\mathbf{E}^{(l+1)} = \sigma \left( \hat{A}\mathbf{E}^{(l)} \mathbf{W}^{(l)}_{1} + \left(\mathbf{E}^{(l)} \odot \hat{A}\mathbf{E}^{(l)} \right) \mathbf{W}^{(l)}_{2} \right)$$

where $\widehat{A}$ denotes the normalized adjacency matrix with self-loops. The first term corresponds to the aggregation of structural information, while the second term represents the semantic–structural synergy component. Through multi-layer iterative propagation, the model is capable of progressively capturing deep cross-modal interaction patterns.

### Feature fusion and prediction

In the global–local feature fusion prediction module, we first concatenate the multi-layer embeddings to obtain the integrated node representation matrix:

17 $$\mathbf{E} = \mathrm{Concat} \left( \mathbf{E}^{(1)}, \mathbf{E}^{(2)}, \ldots, \mathbf{E}^{(l)} \right)$$

where ${E}^{(l)}$ denotes the node embedding obtained after cross-modal propagation at the *l*th layer. The concatenated matrix E thus contains the overall embeddings of both circRNA and drug nodes. Next, we divide E into two subsets:

18 $$\mathbf{E}_{\mathrm{drug}} = \mathbf{E}_{[1:N_{d},:]}$$

19 $$\mathbf{E}_{\mathrm{cir}} = \mathbf{E}_{[N_{d} +1:,:]}$$

where ${E}_{drug}$ represents the embeddings of drug nodes, ${E}_{cir}$​ represents the embeddings of circRNA nodes, and ${N}_{d}$ denotes the number of drug nodes. Meanwhile, for the heterogeneous graph *H*, we conduct semantic and structural feature modeling to obtain the global fusion representation ${E}_{heter}$. To achieve the integration of global and local information, we perform concatenation and linear projection for drug and circRNA embeddings separately:

20 $$\mathbf{C} = \sigma \left(W_{c} \left(\mathbf{E}^{(d)}_{\mathrm{heter}}\|\mathbf{E}_\mathrm{drug}\right)+b_c\right)$$

21 $$\mathbf{D} = \sigma \left(W_{d} \left(\mathbf{E}^{(d)}_{\mathrm{heter}}\|\mathbf{E}_\mathrm{cir}\right)+b_d\right)$$

where ${E}_{heter}^{(d)}$ and ${E}_{heter}^{(c)}$ denote the corresponding subsets of drug and circRNA embeddings extracted from the global representation ${E}_{heter}$, and ‖ indicates the concatenation operation. In this way, C and D simultaneously incorporate global–local fused information.

Finally, we apply the inner product operation followed by a nonlinear activation function $\sigma (\cdot )$ to map the scores into association probabilities between circRNAs and drugs: 

22 $$\widehat{y}_{cd} = \sigma (\mathbf{C\;\;D})$$

## Data Availability

All data generated or analysed during this study are included in this published article, its supplementary information files and publicly available repositories: Zenodo [72] (https://zenodo.org/records/19707579) and GitHub (https://github.com/cc646201081/AGCECDA).

## Abbreviations

circRNA: Circular RNA
miRNA: MicroRNA
RBP: RNA-binding protein
ceRNA: Competing endogenous RNA
GIP: Gaussian interaction profile
CSS: CircRNA sequence similarity
DSS: Drug structure similarity
CS: CircRNA integrated similarity matrix
DS: Drug integrated similarity matrix
CGS: CircRNA GIP similarity matrix
DGS: Drug GIP similarity matrix
GCN: Graph convolutional network
GNN: Graph neural network
NGCF: Neural graph collaborative filtering
DRD: Drug response difference
FDR: False discovery rate
AUC: Area under the curve
AUROC: Area under the receiver operating characteristic curve
AUPR: Area under the precision–recall curve
ACC: Accuracy
CV: Cross-validation
KF: *K*-fold
CCLE: Cancer Cell Line Encyclopedia
GDSC: Genomics of Drug Sensitivity in Cancer
CTRP: Cancer Therapeutics Response Portal
